# Silent Intraoperative Right Coronary Artery Occlusion at a Prior Stent Site Progressing to Ventricular Fibrillation During Hip Hemiarthroplasty: A Case Report

**DOI:** 10.7759/cureus.117610

**Published:** 2026-10-07

**Authors:** Wei Wang, Qun Wan, Zhiying Yu, Zongmei Yang, Lin Ba

**Affiliations:** 1 Operating Room/Nursing, Binzhou People's Hospital, Binzhou, CHN; 2 Standardized Anesthesiology Training, Binzhou People's Hospital, Binzhou, CHN; 3 Anesthesiology, Binzhou People's Hospital, Binzhou, CHN

**Keywords:** acute right coronary artery occlusion, coronary stent, elderly patients, hip fracture, nursing surveillance, perioperative myocardial infarction, spinal anesthesia, ventricular fibrillation

## Abstract

Perioperative myocardial infarction may occur without typical ischemic symptoms in elderly patients undergoing hip fracture repair. We describe the case of an 86-year-old woman with hypertension, coronary artery disease, and prior right coronary artery stenting who underwent right hip hemiarthroplasty under spinal anesthesia after clopidogrel had been withheld for one week. Continuous electrocardiographic monitoring showed progressive ST-segment elevation first recognized by the anesthesia nurse, who verified the finding and immediately informed the anesthesiology team. ST-segment elevation preceded a decline in heart rate and blood pressure and was followed by ventricular fibrillation during emergency transfer for coronary angiography. Repeated cardiopulmonary resuscitation and 200-J defibrillation produced transient rhythm recovery, with recurrent ventricular fibrillation until coronary reperfusion. Angiography showed acute right coronary artery occlusion at the previous stent site with thrombus; balloon angioplasty, intracoronary tirofiban, and implantation of a 2.75 × 29 mm drug-eluting stent restored TIMI grade 3 flow, with approximately 30% residual stenosis. Troponin T was >50 ng/mL on postoperative day 1, 4056 ng/mL on day 5, and 900 ng/mL on day 9; no major bleeding, transfusion, or neurological complications were documented. The patient ambulated before discharge on postoperative day 15. This case illustrates a descriptive temporal sequence in which progressive monitor-detected ST-segment changes preceded hemodynamic deterioration and subsequent ventricular fibrillation, followed by nursing verification, escalation, and multidisciplinary rescue; it does not establish that any specific monitoring or escalation strategy altered the outcome. Because this is a single case, the chronology described is observational and does not permit causal inference regarding the outcome.

## Introduction

Hip fracture surgery in older adults is associated with substantial perioperative cardiovascular risk. A recent systematic review reported a 30-day myocardial infarction (MI) prevalence of approximately 2% after hip fracture surgery, while population-based studies have shown that MI after repair is associated with marked short- and long-term mortality [1,2]. In a retrospective cohort study reported by Gupta et al., 167 of 1,212 elderly patients developed perioperative MI (PMI) within seven days after hip fracture surgery, and 75% of these patients who developed PMI were asymptomatic [3]. 

Patients with previous coronary stent implantation undergoing urgent non-cardiac surgery represent a particularly challenging population. Perioperative interruption of antiplatelet therapy may be required to balance bleeding risk and thrombotic risk, but acute stent thrombosis remains a concern in selected high-risk patients. The mechanism of perioperative stent-site occlusion is multifactorial and may involve underlying coronary disease, stent-related factors, perioperative stress responses, and antiplatelet interruption. Individualized cardiovascular risk assessment and multidisciplinary decision-making are therefore essential.

This diagnostic challenge is amplified during anesthesia and surgery, when pain, stress responses, communication limitations, and rapidly changing hemodynamics may reduce the reliability of symptom-based assessment. Older hip fracture patients with coexisting coronary heart disease are particularly vulnerable to postoperative and perioperative cardiac events [4].

We report the case of an 86-year-old woman with a previous coronary stent who developed progressive intraoperative ST-segment elevation, hemodynamic deterioration, acute inferior myocardial infarction, and ventricular fibrillation during hip hemiarthroplasty under spinal anesthesia. Continuous electrocardiographic and hemodynamic surveillance provided objective evidence of evolving ischemia before overt cardiovascular collapse; however, this observation should not be interpreted as proof that a particular monitoring strategy is effective in all high-risk patients. The educational purpose of this report is to illustrate the chronology of monitor-detected ST-segment changes, nursing verification and escalation, multidisciplinary resuscitation and reperfusion, and post-event surveillance in a single high-risk perioperative case, without inferring efficacy from this clinical experience.

## Case presentation

An 86-year-old woman was admitted after a fall causing a right femoral neck fracture and was scheduled for hemiarthroplasty. Her medical history included hypertension for approximately 30 years, treated with irbesartan and hydrochlorothiazide, and coronary artery disease. She had undergone percutaneous coronary intervention (PCI) six years earlier after presenting with chest tightness, with a stent implanted in the right coronary artery; the type of the original stent and the specific diagnostic indication for that PCI were not available in the accessible records. One week prior to the admission for surgery, clopidogrel had been discontinued. The available records did not document who recommended the interruption, the specific rationale, or whether aspirin was continued preoperatively. Before the fracture, she was independent in activities of daily living and could climb stairs without assistance.

Preoperative electrocardiography showed sinus rhythm with ST-T abnormalities, including ST-segment elevation in leads II, III, and aVF. The available record did not permit a reliable quantitative comparison between these baseline inferior-lead abnormalities and the subsequent intraoperative changes. Echocardiography demonstrated left atrial enlargement, aortic and mitral valve calcification with mild regurgitation, mild-to-moderate tricuspid regurgitation, mild pulmonary hypertension, and a left ventricular ejection fraction of 65%.

At 08:10 hours, spinal anesthesia was performed with 2.0 mL of 0.5% hyperbaric ropivacaine, and the sensory block was fixed at T10 by 08:20. Surgery began at 09:00. At 09:32, the perioperative monitor showed progressive ST-segment elevation. Review of the stored ST-analysis display showed an increase in lead II from approximately 0.17 mV at 09:33:06 to 0.35 mV at 09:37:06 (Figure 1). The reported ST values were transcribed from the monitor's stored ST-analysis display rather than retrospectively re-measured from the printed tracing.

**Figure 1:**
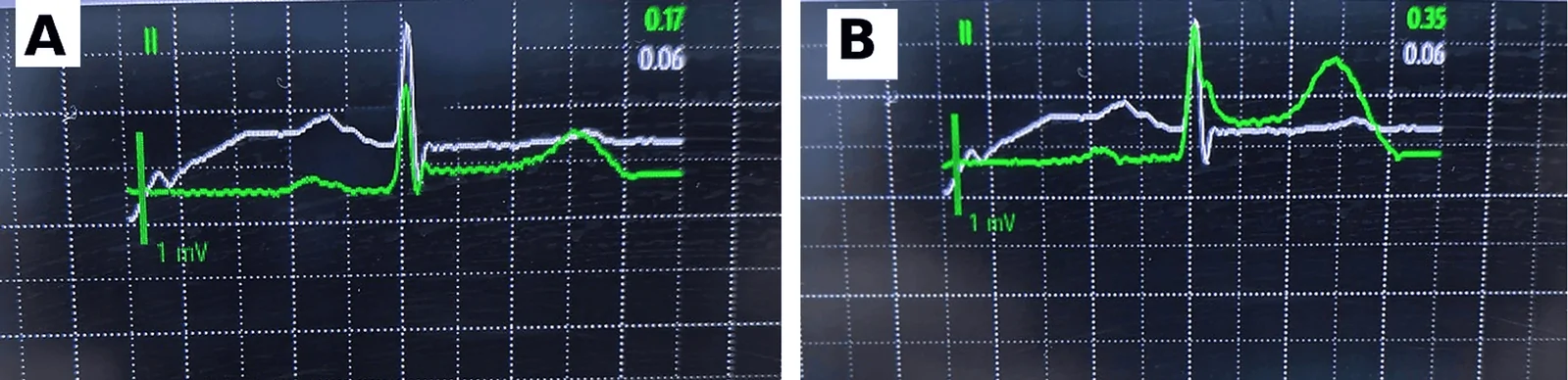
Dynamic intraoperative ST-segment elevation before hemodynamic collapse. (A) Stored ST-analysis strip at 09:33:06 showing lead II ST elevation of approximately 0.17 mV. (B) At 09:37:06, the displayed ST elevation had increased to approximately 0.35 mV.

The anesthesia nurse recognized the abnormal trend, verified the monitor finding, and immediately communicated the concern. Assistance was requested, and urgent cardiology consultation was initiated at approximately 09:33. The cardiologist arrived within approximately 10 minutes and performed an initial assessment over the following 5-10 minutes. By 09:34, heart rate and blood pressure had begun to fall. Intravenous nitroglycerin, norepinephrine, and dopamine were administered, and invasive arterial pressure monitoring was established in the setting of persistent ST-segment abnormalities and hemodynamic deterioration. Coronary spasm was initially considered, but the electrocardiographic abnormalities and hemodynamic instability persisted. An urgent 12-lead ECG was obtained at approximately 10:10-10:15 and interpreted clinically as acute inferior myocardial infarction (Figure 2). The decision to proceed with urgent coronary angiography/PCI was made approximately 5-10 minutes after this diagnosis, and emergency transfer from the operating-room pathway began at approximately 10:40. The exact catheterization-laboratory arrival time was not available.

**Figure 2:**
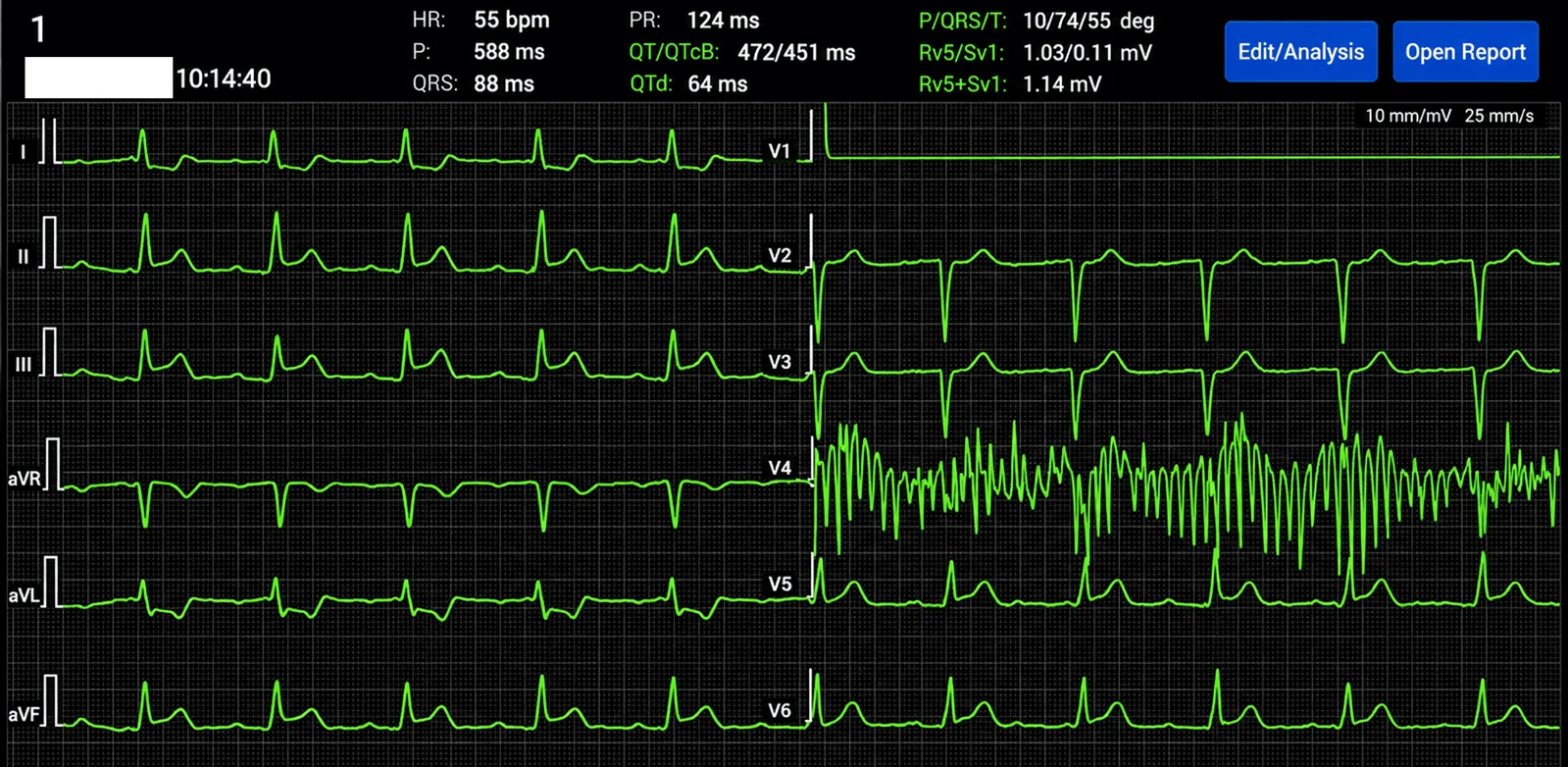
Twelve-lead electrocardiogram obtained during intraoperative deterioration at approximately 10:15 and interpreted clinically as acute inferior myocardial infarction

Between 10:41 and 10:45, ventricular fibrillation occurred during emergency transfer to the catheterization laboratory (Figure 3A). During transfer, nursing staff continued ECG and hemodynamic surveillance, prepared the defibrillator and resuscitation equipment, and assisted with cardiopulmonary resuscitation (CPR) and defibrillation according to team-assigned roles. CPR was performed before each defibrillation attempt, with each cycle lasting approximately one to two minutes, and electrical defibrillation was delivered at 200 J per attempt. Each shock was followed by a brief period of organized rhythm, but ventricular fibrillation recurred after approximately 30-40 seconds until coronary reperfusion. Because rhythm recovery was transient before reperfusion, a single sustained pre-PCI return-of-spontaneous-circulation time could not be assigned from the available monitor sequence; an organized rhythm was documented at approximately 10:56 (Figure 3B). The exact catheterization-laboratory arrival time, coronary angiography start time, and PCI completion time were not retrievable from the accessible records.

**Figure 3:**
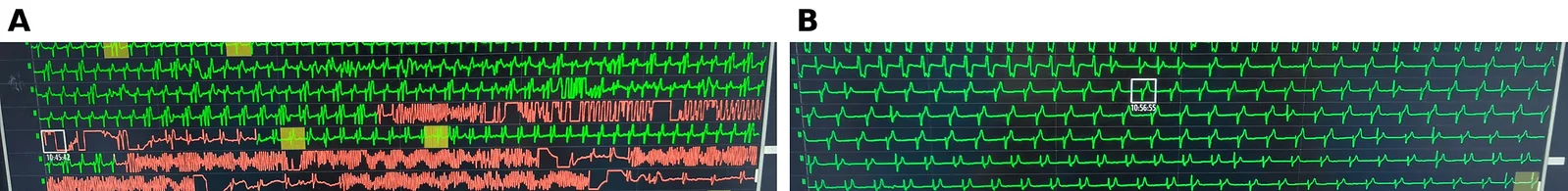
Rhythm deterioration and recovery during emergency transfer and coronary intervention. (A) Monitor review around 10:45, documenting the ventricular fibrillation episode and defibrillation period. (B) Organized rhythm documented at approximately 10:56 after successful electrical defibrillation.

Coronary angiography demonstrated acute proximal-to-mid right coronary artery occlusion at the previous stent site with thrombus. Balloon angioplasty was performed. Because substantial residual stenosis and thrombus persisted after balloon dilation (approximately 80-90% residual mid-segment stenosis with thrombus), intracoronary tirofiban (6 mL) was administered (the concentrated vial presentation was not available), and an additional 2.75 × 29 mm drug-eluting stent was implanted. Final angiography showed TIMI grade 3 flow with approximately 30% residual stenosis at the stent opening/distal edge. Post-PCI antithrombotic treatment included aspirin 300 mg, ticagrelor 180 mg, tirofiban, and intravenous heparin sodium 12,500 U. 

Postoperative cardiac biomarkers documented myocardial injury and subsequent decline. On postoperative day 1, troponin T was >50 ng/mL, CK-MB was 130.51 ng/mL, myoglobin was 400.64 mg/mL, and BNP was 8994.37 ng/mL. On postoperative day 5, troponin T was 4056 ng/mL, myoglobin 76.60 mg/mL, and BNP 3481 ng/mL. On postoperative day 9, troponin T was 900 ng/mL, myoglobin 50.90 mg/mL, and BNP 3187 ng/mL. The exact clock times of these postoperative samples, the troponin T assay methodology, and the laboratory reference range/upper reference limit were not available in the accessible laboratory record. No major bleeding, transfusion, or neurological complications occurred.

**Table 1:** Perioperative timeline of cardiovascular deterioration and rescue PCI: percutaneous coronary intervention; ROSC: return of spontaneous circulation

Time	Clinical event	Immediate nursing/clinical response
08:10	Spinal anesthesia administered	Nursing: continuous ECG and hemodynamic surveillance initiated. Clinical team: anesthesia management and routine intraoperative monitoring.
08:20	Sensory block fixed at T10	Nursing: ongoing surveillance and documentation of vital signs.
09:00	Hemiarthroplasty started	Routine intraoperative monitoring continued.
09:32-09:37	Progressive ST-segment elevation on stored monitor ST-analysis display (lead II approximately 0.17 to 0.35 mV)	Nursing: recognized and verified the abnormal trend and immediately communicated concern. Assistance requested and urgent cardiology consultation initiated at approximately 09:33.
~09:43	Cardiologist arrived within approximately 10 min of consultation; initial assessment lasted approximately 5-10 minutes	Multidisciplinary assessment of evolving ECG and hemodynamic abnormalities.
09:34	Heart rate and blood pressure declined	Clinical team: nitroglycerin, norepinephrine, dopamine, and invasive arterial pressure monitoring initiated. Exact drug doses/rates/durations were unavailable. Nursing: close hemodynamic observation and communication.
10:10-10:15	12-lead ECG obtained and interpreted as acute inferior myocardial infarction	Urgent coronary angiography/PCI considered following cardiology assessment.
~10:15-10:25	Decision for emergency transfer for coronary angiography/PCI (approximately 5-10 minutes after diagnosis)	Nursing: continued ECG/hemodynamic surveillance and prepared defibrillator/resuscitation equipment for transfer.
~10:40	Emergency transfer from operating-room pathway initiated	Nursing: continuous ECG/hemodynamic monitoring, transfer support, and resuscitation readiness.
10:41-10:45	Recurrent ventricular fibrillation during emergency transfer	CPR approximately 1-2 minutes before each defibrillation attempt; 200-J shocks produced transient organized rhythm, with recurrent VF after approximately 30-40 seconds. Nursing assisted with CPR/defibrillation and monitored rhythm/hemodynamics.
~10:56	Organized rhythm documented on monitor	A single sustained pre-PCI ROSC time could not be assigned because rhythm recovery was transient before reperfusion.
Time unavailable	Coronary angiography: acute proximal-to-mid RCA occlusion at prior stent site with thrombus	Balloon angioplasty performed; persistent approximately 80-90% residual mid-segment stenosis with thrombus prompted intracoronary tirofiban and additional stenting.
Time unavailable	Additional PCI completed	2.75 × 29 mm drug-eluting stent implanted. Final TIMI grade 3 flow with approximately 30% residual stenosis. Exact PCI completion time unavailable.
Post-PCI; exact time unavailable	Antiplatelet/antithrombotic therapy	Aspirin 300 mg, ticagrelor 180 mg, tirofiban (recorded volume 6 mL; concentration/mg dose unavailable), and heparin sodium 12,500 U.
11:53	Atrial fibrillation developed	Electrical cardioversion performed successfully.
12:10	Hemodynamics and rhythm stabilized after PCI	Multidisciplinary assessment performed. Hemiarthroplasty resumed after individualized balancing of ischemic and bleeding risks.
12:54-13:12	Consciousness and motor function recovered	Nursing: neurological assessment, extubation-readiness assessment, and postoperative surveillance.
Postoperative day 1	Cardiac biomarkers documented myocardial injury	Troponin T >50 ng/mL; CK-MB 130.51 ng/mL; myoglobin 400.64 mg/mL; BNP 8994.37 ng/mL. Exact sampling clock time and troponin assay/reference range unavailable.
Postoperative day 5	Serial biomarkers decreased	Troponin T 4056 ng/mL; myoglobin 76.60 mg/mL; BNP 3481 ng/mL. Exact sampling clock time unavailable.
Postoperative day 9	Further biomarker decline	Troponin T 900 ng/mL; myoglobin 50.90 mg/mL; BNP 3187 ng/mL. Exact sampling clock time unavailable.
Postoperative day 11	Functional recovery documented	Patient ambulated out of bed independently.
Postoperative day 15	Discharged after clinical stabilization	No major bleeding, transfusion, neurological complications, or recurrent myocardial infarction documented.

## Discussion

In this patient, acute coronary occlusion initially manifested as a monitor-detected electrocardiographic abnormality rather than a symptom-driven diagnosis. Because this is a single case, the sequence is descriptive only; no causal inference can be made regarding whether monitoring or escalation altered the subsequent deterioration or outcome. In the study by Gupta et al., 75% of elderly patients who met that study's criteria for PMI after hip fracture repair were asymptomatic [3]; however, this should not be generalized to all forms of PMI. In the present case, progressive ST-segment elevation was identified before overt hemodynamic deterioration, and ventricular fibrillation occurred subsequently during emergency transfer. This temporal ordering describes what was observed and should not be interpreted as evidence that earlier recognition prevented, delayed, or modified ventricular fibrillation. Prior perioperative observations have likewise described new ST-segment elevation preceding overt hemodynamic instability [5].

In the present case, the anesthesia nurse recognized and verified the progressive ST-segment change, communicated the concern, maintained ECG/hemodynamic surveillance during transfer, and assisted with resuscitation and handover according to team-assigned roles [6]. However, the nursing contribution should be interpreted within the multidisciplinary perioperative response. Definitive decisions regarding vasoactive therapy, coronary intervention, antithrombotic treatment, and resumption of orthopedic surgery remained multidisciplinary clinical decisions rather than independent nursing interventions.

The mechanism underlying acute right coronary artery occlusion at the previous stent site remains uncertain. Current American Heart Association (AHA)/American College of Cardiology (ACC) and European Society of Cardiology (ESC) perioperative guidance supports stepwise, individualized cardiovascular risk assessment and team-based decision-making for patients with complex cardiovascular disease, with perioperative antithrombotic management balancing thrombotic and bleeding risks [7,8]. In patients with prior PCI, these principles include explicit review of antiplatelet therapy and whether perioperative aspirin can be continued when clinically appropriate. In this case, the accessible records did not document whether aspirin was continued, who recommended clopidogrel interruption, or the specific rationale for stopping it; therefore, we cannot assess whether perioperative antiplatelet management followed or deviated from these guideline principles. Perioperative interruption of clopidogrel was a clinically relevant contextual factor; however, this single case cannot establish a causal relationship between antiplatelet interruption and stent-site thrombosis. Acute stent thrombosis is a complex event potentially influenced by underlying coronary artery disease, stent-related characteristics, endothelial responses, perioperative inflammatory and hypercoagulable states, and individual thrombotic susceptibility. Hence, these missing data limit interpretation of the contribution of perioperative antiplatelet management to the angiographic event.

Once ventricular fibrillation occurred, management priorities shifted from diagnostic evaluation to immediate resuscitation and restoration of coronary blood flow. Repeated CPR and electrical defibrillation were required during emergency transfer, followed by urgent coronary angiography and PCI. Published experience with cardiac arrest associated with acute coronary syndromes emphasizes rapid initial care and timely definitive cardiovascular management [9], while contemporary resuscitation guidance emphasizes high-quality CPR, prompt defibrillation for shockable rhythms, and structured post-arrest care [10]. After restoration of TIMI grade 3 flow and clinical stabilization, the decision to resume hemiarthroplasty was taken after multidisciplinary assessment of competing ischemic, bleeding, and immobilization risks and should not be interpreted as a general recommendation for similar patients.

Postoperative surveillance continued within the integrated multidisciplinary pathway, including rhythm/hemodynamic observation, neurological assessment, laboratory follow-up, extubation-readiness assessment, and structured handover. Structured operating-room-to-ICU and postoperative handover processes have been associated with fewer communication omissions and improved handover quality [11,12].

This report has several limitations. As a single case, it cannot establish causal relationships between ECG monitoring, nursing recognition, antiplatelet interruption, escalation, or clinical outcome, and it should be viewed as an illustrative clinical experience rather than evidence of efficacy for a specific monitoring or escalation strategy. Follow-up duration was also limited. Nevertheless, the detailed chronology provides a clinically relevant description of progressive intraoperative ST-segment changes, acute stent-site occlusion, ventricular fibrillation, emergency PCI, and individualized perioperative decision-making in a high-risk patient. Continuous ECG and hemodynamic surveillance may be considered in selected high-risk perioperative patients in accordance with institutional practice and applicable perioperative monitoring guidance; this case alone does not establish a universal requirement.

## Conclusions

In this patient, progressive intraoperative ST-segment changes preceded hemodynamic deterioration and were followed by ventricular fibrillation during emergency transfer. Nursing verification and escalation occurred before multidisciplinary resuscitation and coronary reperfusion. The subsequent course included restoration of TIMI grade 3 coronary flow, no documented major bleeding or neurological complication, recovery of ambulation, and discharge on postoperative day 15. This case illustrates a clinically important temporal sequence but does not establish the effectiveness of a specific monitoring or escalation strategy.

## References

[1] Goh EL, Khatri A, Costa AB (2025). Prevalence of complications in older adults after hip fracture surgery : a systematic review and meta-analysis. Bone Joint J.

[2] Huddleston JM, Gullerud RE, Smither F (2012). Myocardial infarction after hip fracture repair: a population-based study. J Am Geriatr Soc.

[3] Gupta BP, Huddleston JM, Kirkland LL (2012). Clinical presentation and outcome of perioperative myocardial infarction in the very elderly following hip fracture surgery. J Hosp Med.

[4] Ran S, Yu Q, Fu M, Hou Z, Wang Z (2024). Risk factors and prognosis of postoperative acute myocardial infarction in elderly hip fracture patients combined with coronary heart disease. J Orthop Surg Res.

[5] Jain U (1995). Myocardial injury during reoperation for coronary artery bypass surgery. J Cardiothorac Vasc Anesth.

[6] Dunckley M, Quinn T, Dickson R, Jayram R, Wright C, McDonald R (2006). Acute coronary syndrome nurses: perceptions of other members of the health care team. Accid Emerg Nurs.

[7] Thompson A, Fleischmann KE, Smilowitz NR (2024). 2024 AHA/ACC/ACS/ASNC/HRS/SCA/SCCT/SCMR/SVM guideline for perioperative cardiovascular management for noncardiac surgery: a report of the American College of Cardiology/American Heart Association joint committee on clinical practice guidelines. Circulation.

[8] Halvorsen S, Mehilli J, Cassese S (2022). 2022 ESC guidelines on cardiovascular assessment and management of patients undergoing non-cardiac surgery. Eur Heart J.

[9] Muntean C, Pavin D, Mabo P, Kerharo JY, Boulmier D, Mallédant Y, Daubert JC (2005). Cardiac arrest outside hospital: initial and subsequent cardiological management (Article in French). Arch Mal Coeur Vaiss.

[10] Del Rios M, Bartos JA, Panchal AR (2025). Part 1: executive summary: 2025 American Heart Association guidelines for cardiopulmonary resuscitation and emergency cardiovascular care. Circulation.

[11] Marshall AP, Tobiano G, Murphy N (2019). Handover from operating theatre to the intensive care unit: a quality improvement study. Aust Crit Care.

[12] Reine E, Aase K, Raeder J, Thorud A, Aarsnes RM, Rustøen T (2021). Exploring postoperative handover quality in relation to patient condition: a mixed methods study. J Clin Nurs.

